# Toxicological Assessment of Bromochlorophene: Single and Repeated-Dose 28-Day Oral Toxicity, Genotoxicity, and Dermal Application in Sprague–Dawley Rats

**DOI:** 10.3389/fphar.2021.690141

**Published:** 2021-07-14

**Authors:** Hansol Won, Da Hye Jeong, Hyo-Sook Shin, Jin Hee Lee, Jeong Pyo Lee, Jun-Young Yang, Kikyung Jung, Jayoung Jeong, Jae Ho Oh

**Affiliations:** Division of Toxicological Research, National Institute of Food and Drug Safety Evaluation, Ministry of Food and Drug Safety, Osong, South Korea

**Keywords:** bromochlorophene, repeated-dose 28-day oral toxicity study, genotoxicity, preservative, NOAEL

## Abstract

Bromochlorophene (BCP) has shown good properties in sterilization and antibacterial activity and is widely used as a household chemical. We evaluated the genotoxicity, single and repeated-dose 28-day oral toxicity, and dermal application of a BCP suspension in Sprague–Dawley (SD) rats. For the single-dose toxicity study, a dose of 25–1,000 mg per kg of bodyweight (mg/kg b.w.) of BCP was given once orally to SD rats. Mortality and clinical signs were observed and recorded for the first 30 min after treatment, at 4 h post-administration, and then at least once daily for 14 days after administration. For the repeated-dose 28-day toxicity study, the high dose was set at 1,000 mg/kg b.w. and the middle, middle-low, and low dose were set to 500, 250, and 125 mg/kg, respectively. Hematology and biochemistry parameters were examined. Gross pathologic and histopathologic examinations were performed on selected tissues from all animals. A bacterial reverse mutation assay, *in vitro* chromosomal aberration assay, and *in vivo* micronucleus assay were performed to assess genotoxicity-dermal application exposure assessment of BCP in rats. A high oral approximate lethal dose (ALD) of 1,000 mg/kg was observed in the single-dose toxicity test. During the repeated-dose 28-day time period, most animal deaths after administration occurred during the first 3 weeks. The 1,000 mg/kg b.w. oral dose caused the death of six male rats (6/7) and four female rats (4/7). At 500 mg/kg b.w., the female rats showed mortality (1/7). For the biochemistry assays, cholesterol was increased significantly compared to vehicle in both sexes in the 250 and 500 mg/kg groups. Histopathological changes with treatment-related findings were observed in the pancreas in female rats treated with a high dose of BCP compared with the vehicle group. BCP showed no genotoxic effect. These data suggested that the ALD of BCP, estimated as a non-genotoxic substance, was over 1,000 mg/kg b.w. in the single-dose toxicity study, and the NOAEL of BCP was considered to be 250 mg/kg b.w. for male and female rats after repeated oral administration for 28 days under the present study conditions.

## Introduction

Lots of household chemicals are used these days. As quality of life has become more critical, it has become desirable to prevent toxicity. Therefore, life-friendly materials and consumer products are increasingly being consumed, which has also led to an increase in the number of toxicity study cases (Bilal and Iqbal, 2019; Shahi et al., 2019).

To provide information on chemical safety, the Organization for Economic Cooperation and Development (OECD) and the European Chemicals Agency (ECHA) established “the Global Portal to Information on Chemical Substances” (eChemPortal, 2020), a web-based service offering information on chemical properties such as toxicity, exposure, and use (Müller-Ruch et al., 2020). However, some chemicals have no proper toxicological information, even though they have been registered or permitted as a legal substance by authorities.

2-bromo-6-[(3-bromo-5-chloro-2-hydroxyphenyl) methyl]-4-chlorophenol (BCP), a hydrophobic chemical, is used as an anti-microbial agent in household chemicals like cosmetic emulsion, toothpaste, and deodorants (Moran et al., 2005; Stibany et al., 2017) BCP is a registered preservative at concentrations of up to 0.1% in cosmetic products in many countries, including Korea [MFDS (The Ministry of Food and Drug Safety), 2017a]. The various physicochemical characteristics of preservatives are the major determinants of their toxic potential (Lee et al., 2019; Won et al., 2020). The major exposure pathways of preservative are ingestion, inhalation, and absorption into the skin. Exposure of preservatives *via* the oral route has been closely related to the toxic response of tissues and kidney malfunction as well as cardiovascular, gastrointestinal, and hematologic effects (Tade et al., 2018; Lee and Park, 2019).

Several previous studies have focused on the effect of BCP when used for antimicrobial agents, which is known to be a potential plaque inhibitory agent and an effective bactericide. Toothpaste containing BCP has an anti-plaque effect to reduce dental caries in both humans and animals (Moran et al., 2005; Sadeghi and Assar, 2009).

Although BCP is an ingredient of many household products used in everyday life, research is insufficient. The BCP substance is not listed in the Superfund Amendments and Reauthorization Act (SARA) (SARA, 2017), Proposition 65 (OEHHA, 2020), and Carcinogenic categories (NIOSH, 2007; IARC, 2020).

Our investigation aimed to produce toxicity test information necessary for the evaluation of risk assessment. In our study, BCP risk was indicated by a single-dose oral toxicity study, a 28-day repeated-dose oral toxicity study, and genotoxicity study.

## Materials and Methods

### Test Substance

Bromochlorophene (2-bromo-6-[(3-bromo-5-chloro-2-hydroxyphenyl) methyl]-4-chlorophenol); C_13_H_8_Br_2_Cl_2_O_2_, molecular weight 426.9 g/mol, CAS: 15,435-29-7) was obtained from ALFA chemistry (purity 99%, China) and used as received unless otherwise noted. BCP for oral administration or treatment was formulated in 4% ethanol (Manzo et al., 2012; Thackaberry, 2013).

### Animals

Specific pathogen-free Sprague-Dawley rats [Crl: CD (SD)] were purchased from Koatech Inc., Republic of Korea. The animals were acclimated for 7 days after arrival at the laboratory animal facility of the National Institute of Food and Drug Safety Evaluation of the Ministry of Food and Drug Safety (Osong, Korea). The studies were approved by the Institutional Animal Care and Use Committee (IACUC) (2019, approval no. MFDS-19-002). The animals were housed at a temperature of 22 ± 3°C and relative humidity of 50 ± 20%. The animal rooms were maintained under a 12-h light-dark cycle and 10–20 air changes per hour. The toxicity test was performed strictly using “Toxicity Test Standards for Drugs” from the Korean Ministry of Food and Drug Safety (MFDS (The Ministry of Food and Drug Safety), 2017b). It is also referred to as the OECD Guideline No. 407 (OECD, 2008).

### Single Toxicity Experiment

Six-week-old male and female SD rats (*n* = 5–7 per sex and group) were orally treated with BCP. The vehicle group received 4% ethanol solution in the same volume, while the treated group received BCP at a dosage of 1,000, 700, 250, 80, and 25 mg/kg b.w./day. After 7-day acclimatization, rats were treated once. General clinic observations began 4 h after the rats received the drugs, and then this regime continued every 8 h for at least 7 days.

### Repeated-Dose 28-day Toxicity Experiment

Eighty-four rats (6 weeks old) were randomly assigned into four groups (Siven male and seven female rats in each group). The obtained median approximate lethal dose (ALD) of BCP was 1,000 mg/kg b.w./day in rats. The four groups were divided into: high dose (1,000 mg/kg b.w./day), medium dose (500 mg/kg b.w./day and 250 mg/kg b.w./day), low dose (125 mg/kg b.w./day), vehicle control (4% ethanol solution in same volume), and control (not treat). Rats were administered once daily by gavage with BCP suspension at four doses as above (1,000, 500, 250, and 125 mg/kg b.w./day) at 9:00–10:00 a.m. throughout the experiment for 28 continuous days. The investigators held the animal with the left hand and the syringe with the right hand. Then, they kept the head and neck of the rat in a straight line, thereby enabling easy access to the mouth. In Baoding rats, the head must be fixed to avoid the head twisting at will and affecting the operation of gavage. The intragastric needle entered from the angulus oris of the animal to make the mouth and esophagus align. When the intragastric needle reached a depth of approximately 5 cm, the experimenters gently pushed the syringe slowly to administer the dose. If there was no excessive struggle, the drug was injected slowly. If the resistance was small, the full dose could be administered. In the case of rat struggle, the intragastric needle was pulled out, reserved and operated again. The animals’ general clinic observations, body weight, morbidity, and mortality were recorded daily during administration. Additionally, measurements of food consumption and water intake were recorded weekly.

#### Hematology

Blood samples were collected and placed into tubes containing EDTA-K2 for the hematological analyses. At the end of the drug administration period, the hematological index of rats was analyzed. Standard hematological and biochemistry tests were used to determine the hematological parameters, enzymes, substrates, and products of metabolism. The indicators were white blood cell (WBC), red blood cell (RBC), platelet (PLT), neutrophil (NEUT), lymphocyte (LYM), monocyte (MONO), eosinophil (EOS), basophil (BASO), and reticulocyte (Retic) counts; hemoglobin (HGB), hematocrit (HCT), mean corpuscular volume (MCV), mean corpuscular hemoglobin (MCH), and mean corpuscular hemoglobin concentration (MCHC).

#### Serum Biochemistry

Blood samples were collected, centrifuged at 3,000 rpm for 10 min at 4°C, and stored at −20°C. At the end of the drug administration period, the biochemical blood index of rats in each group was analyzed. The primary biochemical indicators were alanine aminotransferase (ALT), aspartate aminotransferase (AST), alkaline phosphatase (ALP), gamma-glutamyl transferase (GGT), blood urea nitrogen (BUN), creatinine (CREA), total protein (TP), albumin (ALB), cholesterol (T-CHOL), glucose (GLU), triglyceride (TG), total bilirubin (T-BIL), direct bilirubin (D-BIL), lactate dehydrogenase (LDH), creatine kinase (CK), uric acid (UA), calcium (Ca), phosphorus (IP), high-density lipoprotein (HDL), and low-density lipoprotein (LDL).

#### Necropsy

At the end of the 28-day toxicity test, SD rats were fasted overnight and underwent general anesthesia with CO_2_. Necropsies were conducted carefully on all the rats, which either died or survived during experiments. Tissues like liver, spleen, heart, kidney (both), adrenal gland, lung, brain, pituitary gland, thymus, urinary bladder, stomach, intestine, testis, ovary, epididymis, uterus, prostate, seminal vesicle, trachea, esophagus, thyroid gland, salivary gland, skin, femur, and Harderian gland nerves were examined for the macroscopic morphology, and then removed quickly, washed in saline, weighed, and kept in 10% neutral buffered formalin (#0151S, BBC Biochemical, Seattle, WA). In addition, the relative weight of each organ (viscera/body ratio) was calculated based on the animal’s body weight according to the following formula: organ weight/body weight on sacrifice day × 100.

#### Histopathology

Tissues were fixed in a 10% formalin solution (# 0151S, BBC Biochemical, Seattle, WA) containing neutral phosphate-buffered saline, trimmed, processed, and embedded in paraffin, and sectioned at 4-μm thickness; sections were stained with hematoxylin and eosin (H&E) according to routine histological techniques. The histopathological changes were then evaluated using light microscopy (Leica DM 3000, Wetzlar, Germany).

### Genotoxicity

#### Bacterial Reverse Mutation Test

The mutagenic potential of BCP was evaluated by a bacterial reverse mutation assay, according to OECD TG471 (OECD, 2020 [22]). The four histidine auxotroph strains of Salmonella typhimurium TA100, TA1535, TA98, and TA1537, along with a tryptophan auxotroph strain of Escherichia coli, WP2 uvrA (pKM101), were used for bacterial reverse mutation testing (Green and Muriel, 1976; Maron and Ames, 1983 [23, 24]). To induce a metabolic activation system, S9 fraction (Molecular toxicology Inc., Lot No. 4230), a mitochondrial fraction of liver homogenated in SD rats, was added with Cofactor-1 (Genogen Co. Ltd, Lot No. 2009608 Ⅰ). The test strains were exposed to the test article using the pre-incubation method. Based on the results of a range-finding test conducted on the test article, dose ranges were determined using the five test strains in both the presence and absence of the metabolic activation system with two plates per dose. In this study, the highest dose was set at 5,000 μg/plate for all test strains, and six-serial diluted concentrations (5,000, 1,250, 313, 78.1, 19.5, and 4.88 μg/plate) were tested in the main study. The colonies were counted using an automated colony counter.

#### *In vitro* Chromosomal Aberration Test

The *in vitro* chromosomal aberration test was performed according to OECD TG473 (OECD, 2016a). Chinese hamster lung cells (CHL/IU) were obtained from the American Type Culture Collection (ATCC, United States). The cells were maintained with Eagle’s minimum essential medium supplied with 10% fetal bovine serum. The cells were cultured at 37 ± 1°C and 5% CO_2_-95% air using a humidified incubator and cell culture flasks (culture surface 75 cm^2^). The cytotoxicity dose ranges of this study were determined by calculating the relative population of doubling (RPD) in substance-treated cultures observed in a dose range-finding test. The RPD value was determined using the following formula.

$$RPD\;\left(\text{\%}\right)=\frac{\left(No.\;of\;population\;doublings\;in\;treated\;cultures\right)}{\left(No.\;of\;population\;doublings\;in\;control\;cultures\right)}\times 100$$

population doubling = [log (post-treatment cell number/Initial cell number)]/log 2

According to the RPD results of the dose range-finding test, the highest dose was chosen to produce <50% in this test. The assay consisted of short-term (6 h) and continuous (24 h) treatments. Approximately 22 h after the start of the treatment, colcemid was added to each culture at a final concentration of 0.2 μg/ml. The slides of CHL cells were prepared following the hypotonic-methanol-glacial acetic acid-flame drying Giemsa schedule for metaphase plate analysis. More than 300 metaphases were selected and analyzed for each treatment group under ×1,000 magnification using a light microscope. When a significant difference was confirmed after statistical analyses to determine chromosomal aberrations, it was positive.

#### *In vivo* Mammalian Erythrocyte Micronucleus Test

Male 6-week-old CrljOri: CD1(ICR) mice were purchased from Samtako Laboratory Animal, Inc. (Gyeonggi-do, Korea) and were used for experiments at 8 weeks of age after acclimatization for 1 week. The animals were housed in polycarbonate cages. An ambient temperature of 22 ± 3°C, a relative humidity of 50 ± 10%, and a photoperiod of 12 h were maintained throughout the study. All animals used in this study were cared for in accordance with the principles outlined in the “Guide for the Care and Use of Laboratory Animals,” a NIH publication. ICR mice were divided into five groups (*n* = 5) on the basis of their body weights. BCP and negative control was orally administered twice every 24 h to mice at doses of 0, 62.5, 125, and 250 mg/kg. Mitomycin C (MMC) (Lot no. SLBX4310, Sigma-Aldrich), positive control, was administered once intraperitoneally at 2 mg/kg 24 h before sacrifice. The bone marrow cells were then centrifuged at 4°C and 1,000 rpm for 5 min and smeared on a clean slide glass. Smeared slides were air-dried, fixed in methanol, and then stained with 3% Giemsa solution for 30 min. Additionally, the stained slides were observed under the fluorescence microscope at 600x ∼ ×1,000 magnification (B × 51, Olympus, Japan). The number of polychromatic erythrocytes (PCEs), micronucleated polychromatic erythrocytes (MNPCEs), and normochromic erythrocytes (NCEs) among the red blood cells (RBCs, PGE + NCE) were counted. A total of 4,000 PCEs were scored per animal by the same observer for determining the frequencies of micronucleated polychromatic erythrocytes (MNPCEs). PCE/(PCE + NCE) ratio was calculated by counting 500 cells. This animal study was conducted in accordance with OECD guideline for testing chemicals No. 474 (OECD, 2016b) and approval of the Institute Animal Care and Use Committees of Hoseo University [Approval No.: HSIACUC-20-061(1)].

### Dermal Application Study

A percutaneous absorption study was conducted in rats in accordance with the OECD guidelines for an *in vivo* skin absorption test (OECD, 2004). Approximately 12 h prior to experimentation, rats were anaesthetized by isoflurane, and the dorsal skin covering an area of 6 × 6 cm^2^ was shaved with an electric clipper (Electric clipper Model 808, Daito Electric co., Osaka, Japan). The shaved skin surface was gently wiped with acetone to remove sebum. After overnight fasting, each formulation was applied to the shaved dorsal skin of rats covering an area of 4 × 4 cm^2^ (*n* = 5 per each formulation). The applied amount of each formulation was 1% of BCP, and the applied BCP dose was 0.262 mg/kg. At 12 h after topical application, the applied area was softly rinsed off with acetone to remove the remaining formulation on the skin. Blood samples (0.2 ml each) were collected from the jugular vein at 15, 30 min, and 1, 1.5, 2, 4, 6, 8, 10, 12, 24, 36, and 48 h after topical application. Plasma samples were harvested by centrifugation at 4,000 *g* for 10 min and stored at −70°C until analysis. These parameters included terminal half-life (t1/2), the area under the plasma concentration-time curve from time zero to the last observation time point (AUCall) and to infinity (AUCinf), apparent clearance (CL/F), apparent volume of distribution (Vz/F), and mean residence time (MRT). Maximum plasma concentration (Cmax) and time to reach Cmax (Tmax) were obtained directly from the observed data. The topical bioavailability (F) was calculated as F (%) = 100 (AUCtopical × Dose i.v.)/(AUCi.v.xDosetopical).

### Statistical Analysis

In the toxicity study, data were expressed as mean value and standard deviations (mean ± SD). Statistical analyses containing body weight, food consumption, water intake, organ weight, blood biochemical, and biochemistry data were performed by one-way analysis of variance (ANOVA) followed by post hoc Dunnett’s multiple comparison test using GraphPad Prism 5.0 (GraphPad Prism, San Diego, United States). A value of *p* < 0.05 was considered significant. The dermal application was normally distributed and analyzed using the Student’s t-test and one-way analysis of variance (ANOVA). Fisher’s exact test was used for the chromosome aberration test to compare the frequency of aberrant cells between negative control vs treated groups or negative control vs positive control. For the frequencies of micronuclei, the Kastenbaum and Bowman test was used.

## Results

### Single Toxicity

There were no unscheduled deaths. There were no test article-related clinical signs or body weight changes. BCP at a dose of 1,000 mg/kg had no adverse effect on the behavioral responses of the tested rats after 14 days of observation. Physical observations indicated no signs of changes in the skin, fur, eyes mucous membrane, behavior patterns, tremors, salivation, and diarrhea of the rats. There was no mortality observed at the tested dose, nor was weight loss seen in the rats (Table 1). Thus, the ALD of BCP was higher than 1,000 mg/kg in male and female rats (Table 1).

**TABLE 1:** The mortality of rats during the single-dose toxicity study.

Sex	Group/dose (mg/kg)	Number dosed	Body weight (mean ± SD)			Mortality (dead/total)
			Day 0	Day 7	Day 14	
Male	1 (N.C.)	5	165.53 ± 5.10	224.06 ± 5.75	278.63 ± 8.57	0% (0/5)
	2 (0)	7	165.71 ± 6.34	222.58 ± 9.65	275.34 ± 14.40	0% (0/7)
	3 (25)	7	165.35 ± 5.64	221.33 ± 8.56	276.09 ± 13.06	0% (0/7)
	4 (80)	7	165.89 ± 5.24	222.27 ± 6.91	272.71 ± 15.62	0% (0/7)
	5 (250)	7	165.59 ± 5.84	223.09 ± 9.58	275.77 ± 10.92	0% (0/7)
	6 (700)	7	165.82 ± 5.17	223.38 ± 5.67	272.62 ± 10.95	0% (0/7)
	7 (1,000)	7	165.53 ± 6.25	219.89 ± 12.40	278.39 ± 16.28	0% (0/7)
Female	1 (N.C.)	5	128.25 ± 5.23	155.36 ± 9.48	177.57 ± 12.19	0% (0/5)
	2 (0)	7	130.73 ± 6.79	160.55 ± 10.46	184.34 ± 12.21	0% (0/7)
	3 (25)	7	130.68 ± 4.14	163.87 ± 6.42	187.61 ± 9.87	0% (0/7)
	4 (80)	7	130.48 ± 3.59	156.18 ± 9.76	186.56 ± 9.92	0% (0/7)
	5 (250)	7	130.48 ± 3.61	158.68 ± 5.00	180.40 ± 5.33	0% (0/7)
	6 (700)	7	130.35 ± 3.63	153.56 ± 10.11	185.31 ± 3.92	0% (0/7)
	7 (1,000)	7	130.12 ± 5.81	157.27 ± 6.37	177.76 ± 7.76	0% (0/7)

CON, control; VEH, vehicle administered 4% ethanol; data are expressed as means ± SD (*n* = 5–7/group).

Statistics: One-way analysis of variance (ANOVA test) followed by the Dunnett’s test.

### Repeated-Dose 28-Day Toxicity

#### Clinical Signs, Necropsy Findings, and Food and Water Consumption

**FIGURE 1:**
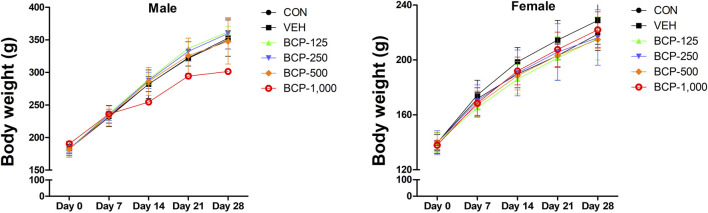
Body weight [mean ± Sprague-Dawley (SD)] of male and female rats treated with 0, 125, 250, 500, and 1,000 mg/kg/day in the 28-day repeated-dose oral toxicity study of Bromochlorophene (BCP). *Significantly different from the vehicle control at *p* < 0.05.

**FIGURE 2:**
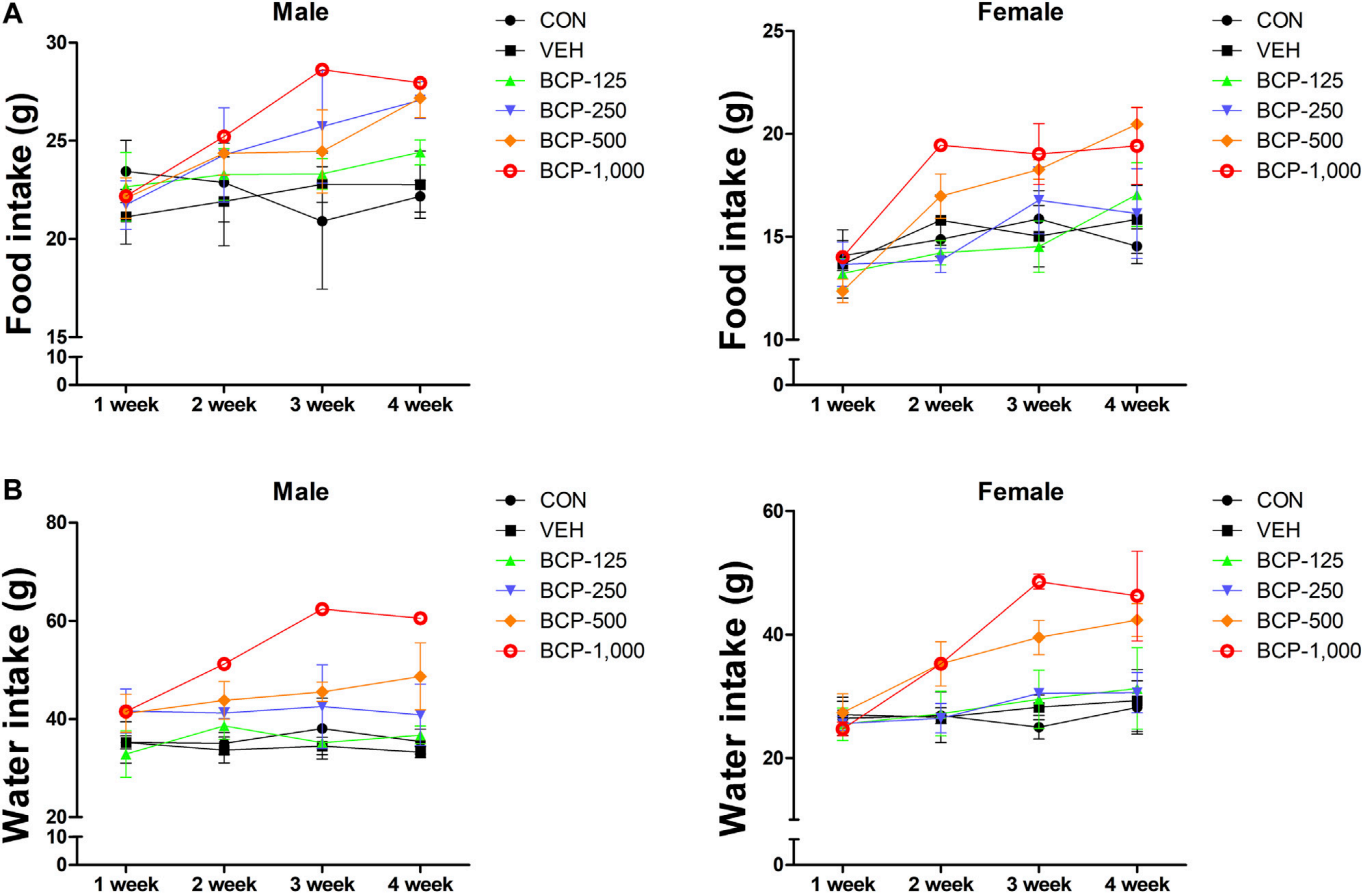
Effect of BCP on the weekly food and water consumption after oral administration in male and female rats for 28 days. A, weekly food consumption. B, weekly water consumption. Data are expressed as the mean ± SD.

Daily oral administration of BCP for 28 days did not induce any obvious symptom of toxicity in rats of both sexes, including the highest dose tested at 1,000 mg/kg body weight. Most of the animal deaths after administration were observed during the first 3 weeks. The mortality rates were 85% (male) and 57% (female) at 1,000 mg/kg (Table 2). Body weight changes were observed during the study period in male treated groups when compared with the vehicle group (Figure 1). No significant differences in body weight were observed among the groups of female rats (*p* > 0.05). Decreased body weight occurred within 2 weeks after BCP administration in a male rat in the 1,000 mg/kg group. However, statistical results are not available as this occurred in one male rat. Food consumption was increased significantly in males in the 125, 250, and 500 mg/kg groups compared to the vehicle group. Food consumption was decreased in female rats from the 125 mg/kg and 250 mg/kg groups at 2 weeks, increased in the 500 mg/kg group at 3–4 weeks, and increased in the 1,000 mg/kg group at 2–4 weeks in comparison with the vehicle group. Water consumption was increased significantly in males in the 250 and 500 mg/kg groups compared to the vehicle group from 1 to 4 weeks after administration. Female rats in the 500 and 1,000 mg/kg groups consumed more water than the control group at 2–4 weeks (Figure 2).

**TABLE 2:** The death of rats during the 28-day dose toxicity study.

Group	Number dosed	Dosage (mg/kg)	Mortality rates (%)	Died (n)	Survived (n)
Male					
1	7	0	0	0	7
2	7	125	0	0	7
3	7	250	0	0	7
4	7	500	0	0	7
5	7	1,000	85	6	1
Female					
1	7	0	0	0	7
2	7	125	0	0	7
3	7	250	0	0	7
4	7	500	17	1	6
5	7	1,000	57	4	3

### Relative Organ Weights of Male and Female Rats

#### Relative Organ Weights

There were generally no significant differences observed in the relative organ weights in this study (Table 3). However, significant differences (*p* < 0.05) were seen in the liver in the male and female treated groups compared with the vehicle group. The relative weight of both kidneys in females in the 500 and 1,000 mg/kg groups were significantly increased (*p* < 0.05).

**TABLE 3:** Toxicity of BCP on percentage of relative organ weight in male and female rats.

Organ (g/100 g body weight)	Groups (mg/kg/day)				
	0	125	250	500	1,000
Male					
Liver	2.92 ± 0.21	2.97 ± 0.19	3.26 ± 0.20^a^	3.46 ± 0.29^a^	3.31 ± 0.00
Kidney-R	0.36 ± 0.02	0.36 ± 0.02	0.38 ± 0.02	0.38 ± 0.04	0.33 ± 0.00
Kidney-L	0.35 ± 0.03	0.36 ± 0.03	0.38 ± 0.02	0.37 ± 0.04	0.34 ± 0.00
Adrenal gland-R (mg)	7.85 ± 0.73	7.91 ± 1.03	8.61 ± 0.33	8.66 ± 1.31	8.99 ± 0.00
Adrenal gland-L (mg)	8.09 ± 1.13	7.78 ± 1.10	8.46 ± 0.82	8.91 ± 1.54	8.69 ± 0.00
Heart	0.42 ± 0.04	0.39 ± 0.03	0.40 ± 0.03	0.39 ± 0.03	0.37 ± 0.00
Lung	0.48 ± 0.04	0.48 ± 0.04	0.49 ± 0.03	0.49 ± 0.04	0.45 ± 0.00
Brain	0.57 ± 0.03	0.56 ± 0.02	0.56 ± 0.03	0.56 ± 0.04	0.59 ± 0.00
Pituitary gland	3.27 ± 0.22	3.37 ± 0.59	3.53 ± 0.73	2.98 ± 0.31	2.75 ± 0.00
Spleen	0.22 ± 0.02	0.23 ± 0.02	0.23 ± 0.04	0.23 ± 0.02	0.18 ± 0.00
Thymus	0.17 ± 0.02	0.17 ± 0.02	0.15 ± 0.02	0.15 ± 0.02	0.11 ± 0.00
Testis-R	0.56 ± 0.05	0.55 ± 0.03	0.59 ± 0.05	0.58 ± 0.05	0.61 ± 0.00
Testis-L	0.55 ± 0.05	0.55 ± 0.03	0.58 ± 0.06	0.58 ± 0.05	0.61 ± 0.00
Epididymides-R	0.15 ± 0.01	0.14 ± 0.01	0.16 ± 0.02	0.15 ± 0.02	0.15 ± 0.00
Epididymides-L	0.15 ± 0.01	0.14 ± 0.01	0.15 ± 0.02	0.15 ± 0.01	0.15 ± 0.00
Prostate	0.15 ± 0.07	0.13 ± 0.03	0.14 ± 0.02	0.12 ± 0.01	0.09 ± 0.00
Seminal vesicle	0.46 ± 0.05	0.41 ± 0.05	0.43 ± 0.05	0.41 ± 0.08	0.42 ± 0.00
Salivary gland	0.20 ± 0.01	0.20 ± 0.02	0.20 ± 0.01	0.19 ± 0.02	0.19 ± 0.00
Thyroid gland-R (mg)	3.52 ± 0.47	3.88 ± 0.42	3.23 ± 0.38	3.34 ± 0.67	2.26 ± 0.00
Thyroid gland-L (mg)	3.33 ± 0.97	3.08 ± 0.40	3.12 ± 0.64	3.22 ± 0.87	2.52 ± 0.00
Female					
Liver	2.67 ± 0.11	2.91 ± 0.14^a^	3.03 ± 0.16^a^	3.46 ± 0.15^a^	3.74 ± 0.15^a^
Kidney-R	0.35 ± 0.02	0.37 ± 0.02	0.37 ± 0.02	0.40 ± 0.03^a^	0.40 ± 0.01^a^
Kidney-L	0.36 ± 0.02	0.36 ± 0.02	0.37 ± 0.02	0.41 ± 0.03^a^	0.40 ± 0.01^a^
Adrenal gland-R (mg)	14.98 ± 1.27	15.75 ± 1.70	14.37 ± 1.79	15.20 ± 1.29	14.99 ± 2.00
Adrenal gland-L (mg)	15.26 ± 2.60	14.71 ± 1.10	14.65 ± 3.22	14.87 ± 0.87	15.47 ± 0.97
Heart	0.42 ± 0.02	0.43 ± 0.03	0.41 ± 0.02	0.43 ± 0.03	0.42 ± 0.03
Lung	0.56 ± 0.03	0.57 ± 0.05	0.58 ± 0.02	0.55 ± 0.03	0.55 ± 0.02
Brain	0.80 ± 0.04	0.82 ± 0.05	0.85 ± 0.05	0.81 ± 0.03	0.80 ± 0.02
Pituitary gland	5.17 ± 1.57	5.35 ± 0.85	4.85 ± 0.38	4.89 ± 0.62	4.60 ± 0.72
Spleen	0.27 ± 0.02	0.28 ± 0.03	0.28 ± 0.01	0.29 ± 0.05	0.25 ± 0.02
Thymus	0.17 ± 0.02	0.16 ± 0.02	0.18 ± 0.03	0.15 ± 0.04	0.15 ± 0.01
Ovary-R	22.22 ± 3.68	22.28 ± 3.16	23.86 ± 5.54	22.56 ± 4.04	28.50 ± 10.81
Ovary-L	22.82 ± 1.64	22.23 ± 3.35	24.51 ± 4.55	22.61 ± 4.97	28.93 ± 9.99
Uterus	0.20 ± 0.02	0.32 ± 0.25	0.22 ± 0.07	0.29 ± 0.16	0.22 ± 0.04
Salivary gland	0.21 ± 0.02	0.21 ± 0.01	0.22 ± 0.01	0.21 ± 0.02	0.23 ± 0.03
Thyroid gland-R (mg)	4.16 ± 1.01	3.96 ± 1.02	4.54 ± 1.23	4.12 ± 0.91	3.60 ± 0.70
Thyroid gland-L (mg)	4.08 ± 0.74	4.73 ± 2.20	4.68 ± 1.00	3.50 ± 0.69	3.03 ± 0.73

R, right; L, left. Data are expressed as means ± SD (*n* = 7/group).

a Significantly different from vehicle control group (*p* < 0.05).

#### Hematology and Serum Biochemistry

The adverse effects of 28-day repeated administration of BCP on hematological and serum biochemistry are presented in Tables 4, 5. In the case of males, hemoglobin (HGB) and hematocrit (HCT) were increased (*p* < 0.05) in the 125 mg/kg group compared to the vehicle group. Red blood cells (RBC), HGB, and HCT were significantly (*p* < 0.05) increased in the 500 mg/kg group compared to the vehicle group. In the 1,000 mg/kg group of females, MCV was significantly (*p* < 0.05) increased, and MCHC was decreased compared to the vehicle group.

**TABLE 4:** Hematological parameter of BCP in male and female rats.

Parameter	Groups (mg/kg/day)				
	0	125	250	500	1,000
Male					
WBC (× 10^3^ cells/μl)	7.2 ± 1.5	9.2 ± 1.3	7.3 ± 2.5	8.7 ± 2.5	6.6 ± 0.0
RBC (× 10^6^ cells/μl)	7.9 ± 0.4	8.3 ± 0.3	8.1 ± 0.2	8.3 ± 0.3^a^	9.1 ± 0.0
HGB (g/dL)	14.9 ± 0.8	16.0 ± 0.5^a^	15.3 ± 0.4	16.0 ± 0.5^a^	17.5 ± 0.0
HCT (%)	46.5 ± 2.7	49.4 ± 1.2^a^	47.8 ± 1.3	49.9 ± 1.5^a^	54.9 ± 0.0
MCV (fL)	58.8 ± 1.6	59.7 ± 1.1	59.0 ± 1.2	59.8 ± 0.5	60.4 ± 0.0
MCH (pg)	18.8 ± 0.8	19.3 ± 0.3	18.9 ± 0.4	19.2 ± 0.3	19.3 ± 0.0
MCHC (g/dL)	32.0 ± 1.0	32.3 ± 0.4	32.1 ± 0.4	32.1 ± 0.3	31.9 ± 0.0
PLT (x 10^3^ cells/μl)	1,044.6 ± 58.8	1,009.3 ± 39.7	901.9 ± 376.8	931.3 ± 120.4	727.0 ± 0.0
NEUT (% of WBC)	10.4 ± 1.1	9.2 ± 2.9	11.2 ± 3.4	10.1 ± 2.3	5.8 ± 0.0
LYM (% of WBC)	83.9 ± 1.5	85.1 ± 2.8	83.4 ± 3.9	84.0 ± 2.6	90.5 ± 0.0
MONO (% of WBC)	3.4 ± 1.0	3.5 ± 0.6	3.1 ± 0.9	3.2 ± 0.9	1.9 ± 0.0
EOS (% of WBC)	0.7 ± 0.2	0.5 ± 0.1	0.6 ± 0.4	0.6 ± 0.2	0.2 ± 0.0
BASO (% of WBC)	0.5 ± 0.1	0.7 ± 0.2	0.7 ± 0.2	0.7 ± 0.2	0.6 ± 0.0
Retic (%)	2.5 ± 0.3	2.7 ± 0.4	2.6 ± 0.3	2.4 ± 0.4	1.9 ± 0.0
Female					
WBC (x 10^3^ cells/μl)	5.8 ± 1.3	6.6 ± 2.3	4.7 ± 1.6	4.6 ± 0.8	4.7 ± 1.1
RBC (× 10^6^ cells/μl)	8.0 ± 0.2	8.0 ± 0.5	7.8 ± 0.2	7.8 ± 0.4	7.7 ± 0.4
HGB (g/dL)	15.1 ± 0.3	15.2 ± 0.8	14.8 ± 0.5	15.1 ± 0.6	15.1 ± 0.7
HCT (%)	45.5 ± 1.1	45.9 ± 2.8	44.6 ± 1.7	45.8 ± 1.9	46.9 ± 1.7
MCV (fL)	57.3 ± 1.3	57.4 ± 1.8	57.2 ± 1.3	58.9 ± 1.3	60.9 ± 1.2^a^
MCH (pg)	19.0 ± 0.3	19.0 ± 0.6	19.0 ± 0.3	19.4 ± 0.7	19.6 ± 0.3
MCHC (g/dl)	33.2 ± 0.5	33.1 ± 0.5	33.2 ± 0.4	32.9 ± 0.5	32.2 ± 0.3^a^
PLT (× 10^3^ cells/μl)	1,101.4 ± 68.8	1,048.7 ± 59.8	1,056.0 ± 88.7	1,093.8 ± 447.1	1,012.3 ± 16.3
NEUT (% of WBC)	12.4 ± 4.1	20.2 ± 25.6	10.5 ± 3.5	12.5 ± 8.5	17.9 ± 5.3
LYM (% of WBC)	82.8 ± 4.0	72.7 ± 28.9	85.1 ± 3.2	82.8 ± 9.7	78.9 ± 5.7
MONO (% of WBC)	2.5 ± 0.7	4.9 ± 4.4	2.3 ± 0.2	2.7 ± 1.2	1.8 ± 0.6
EOS (% of WBC)	0.9 ± 0.4	0.9 ± 0.8	0.8 ± 0.3	0.6 ± 0.2	0.4 ± 0.1
BASO (% of WBC)	0.5 ± 0.1	0.5 ± 0.2	0.5 ± 0.1	0.6 ± 0.2	0.5 ± 0.3
Retic (%)	2.3 ± 0.4	2.3 ± 0.8	1.9 ± 0.3	2.2 ± 0.5	2.8 ± 0.4

WBC, white blood cell count; RBC, red blood cell count; HCB, hemoglobin; HCT, hematocrit; MCV, mean corpuscular volume; MCH, mean corpuscular hemoglobin; MCHC, mean corpuscular hemoglobin concentration; PLT, platelet count; NEUT, neutrophil; LYM, lymphocyte; MONO, monocyte; EOS, eosinophil; BASO, basophil; Retic, reticulocyte. Data are expressed as means ± SD (*n* = 7/group).

a Significantly different from vehicle control group (*p* < 0.05).

**TABLE 5:** Clinical biochemistry parameter of BCP in male and female rats.

Parameter	Groups (mg/kg/day)				
	0	125	250	500	1,000
Male					
ALT (U/L)	37.7 ± 2.3	38.7 ± 12.9	60.6 ± 21.1*	47.6 ± 11.5	50.0 ± 0.0
AST (U/L)	96.0 ± 18.7	113.4 ± 14.5	119.6 ± 43.8	88.9 ± 15.9	117.0 ± 0.0
ALP (U/L)	164.4 ± 27.4	159.1 ± 20.0	152.9 ± 13.0	138.3 ± 14.8	138.3 ± 14.8
GGT (U/L)	3.2 ± 1.1	2.8 ± 0.8	2.7 ± 0.6	3.8 ± 0.4	4.0 ± 0.0
BUN (mg/dL)	17.0 ± 2.5	20.2 ± 3.3	19.4 ± 3.8	20.2 ± 2.3	25.0 ± 0.0
CREA (mg/dL)	0.6 ± 0.1	0.6 ± 0.1	0.6 ± 0.1	0.7 ± 0.1	0.6 ± 0.0
TP (g/L)	5.6 ± 0.2	5.8 ± 0.2	5.9 ± 0.3*	5.8 ± 0.3	5.9 ± 0.0
ALB (g/dL)	3.2 ± 0.1	3.3 ± 0.1	3.4 ± 0.1	3.3 ± 0.2	3.3 ± 0.0
T-CHO (mg/dL)	74.1 ± 10.2	75.3 ± 13.7	99.7 ± 13.5*	99.7 ± 21.0*	120.0 ± 0.0
GLU (mg/dL)	166.9 ± 49.9	141.7 ± 10.9	153.9 ± 25.6	162.4 ± 24.9	235.0 ± 0.0
TG (mmol/L)	56.4 ± 24.9	46.3 ± 11.8	54.9 ± 16.1	53.7 ± 14.7	46.0 ± 0.0
T-BIL (mg/dL)	0.2 ± 0.0	0.2 ± 0.0	0.2 ± 0.0	0.2 ± 0.0	0.2 ± 0.0
D-BIL (mg/dL)	0.7 ± 0.0	0.7 ± 0.0	0.6 ± 0.4	0.2 ± 0.4*	0.0 ± 0.0
LDH (U/L)	1,379.4 ± 725.3	1,460.6 ± 580.4	1,184.3 ± 781.7	1,045.1 ± 560.0	1870.0 ± 0.0
CK (U/L)	563.6 ± 316.5	820.0 ± 296.0	414.9 ± 171.6	398.1 ± 152.5	1,030.0 ± 0.0
UA (mg/dL)	1.6 ± 0.4	1.4 ± 0.2	1.5 ± 0.3	1.3 ± 0.3	1.1 ± 0.0
CA (mg/dL)	9.6 ± 0.6	9.8 ± 0.4	9.8 ± 0.2	9.9 ± 0.4	9.4 ± 0.0
IP (mg/dL)	9.2 ± 0.8	9.2 ± 0.6	9.0 ± 1.0	9.4 ± 0.8	8.1 ± 0.0
HDL (mg/dL)	64.3 ± 9.9	66.1 ± 9.2	82.9 ± 12.0*	83.3 ± 18.3*	105.0 ± 0.0
LDL (mg/dL)	15.1 ± 5.0	16.4 ± 6.9	21.9 ± 3.5	22.4 ± 7.0	24.0 ± 0.0
Female					
ALT (U/L)	41.3 ± 12.2	34.0 ± 4.5	32.0 ± 7.3	63.5 ± 75.6	59.7 ± 10.0
AST (U/L)	104.1 ± 33.1	86.6 ± 15.6	84.7 ± 14.2	142.0 ± 117.2	109.0 ± 24.3
ALP (U/L)	115.3 ± 17.3	114.0 ± 26.5	94.4 ± 15.7	120.5 ± 16.9	140.7 ± 42.8
GGT (U/L)	2.4 ± 1.3	1.0 ± 1.0*	4.4 ± 0.8*	4.7 ± 0.8*	4.0 ± 1.0
BUN (mg/dL)	17.7 ± 3.1	16.7 ± 2.5	20.2 ± 3.8	24.0 ± 5.7*	25.6 ± 6.4*
CREA (mg/dL)	0.6 ± 0.0	0.6 ± 0.1	0.6 ± 0.1	0.6 ± 0.1	0.6 ± 0.1
TP (g/L)	5.7 ± 0.2	5.7 ± 0.2	6.0 ± 0.1*	6.0 ± 0.2*	6.3 ± 0.2*
ALB (g/dL)	3.3 ± 0.1	3.3 ± 0.1	3.4 ± 0.1	3.4 ± 0.2	3.5 ± 0.1 *
T-CHO (mg/dL)	87.7 ± 13.0	110.3 ± 9.3*	107.4 ± 13.1*	131.8 ± 18.2*	155.7 ± 8.5*
GLU (mg/dL)	129.9 ± 9.0	136.9 ± 27.4	151.0 ± 24.5	156.7 ± 10.0	136.7 ± 15.0
TG (mmol/L)	37.4 ± 13.4	56.9 ± 24.2	90.6 ± 27.9*	83.3 ± 53.6*	91.3 ± 13.6
T-BIL (mg/dL)	0.2 ± 0.0	0.2 ± 0.0	0.2 ± 0.1	0.2 ± 0.1	0.2 ± 0.1
D-BIL (mg/dL)	0.0 ± 0.0	0.0 ± 0.0	0.0 ± 0.0	0.0 ± 0.0	0.0 ± 0.0
LDH (U/L)	1,147.7 ± 382.2	1,192.7 ± 232.6	1,115.6 ± 547.6	1,360.3 ± 731.7	1,260.7 ± 448.2
CK (U/L)	293.6 ± 76.5	267.7 ± 100.3	279.1 ± 64.7	313.5 ± 136.3	350.0 ± 142.3
UA (mg/dL)	1.3 ± 0.4	1.3 ± 0.2	1.2 ± 0.3	1.3 ± 0.5	1.3 ± 0.2
CA (mg/dL)	9.5 ± 0.3	9.7 ± 0.4	9.6 ± 0.4	9.7 ± 0.6	10.1 ± 0.2
IP (mg/dL)	7.7 ± 1.4	7.7 ± 1.1	6.9 ± 0.6	8.6 ± 0.9	8.8 ± 0.9
HDL (mg/dL)	75.6 ± 9.0	88.7 ± 2.6*	86.0 ± 7.9	105.7 ± 11.7*	120.7 ± 13.5*
LDL (mg/dL)	12.6 ± 4.4	15.0 ± 3.0	16.1 ± 4.6	20.5 ± 4.4*	23.3 ± 4.2*

ALT, alanine aminotransferase; AST, aspartate aminotransferase; ALP, alkaline phosphatase; GGT, gamma-glutamyl transferase; BUN, blood urea nitrogen; CREA, creatinine; TP, total protein; ALB, albumin; T-CHOL, cholesterol; GLU, glucose; TG, triglyceride; T-BIL, total bilirubin; D-BIL, direct bilirubin; LDH, lactate dehydrogenase; CK, creatine kinase; UA, uric acid; CA, calcium; IP, phosphorus; HDL, high density lipoprotein; LDL, low density lipoprotein. Data are expressed as means ± SD (*n* = 7/group). *Significantly different from vehicle control group (*p* < 0.05).

Cholesterol was increased (*p* < 0.05) in both sexes in the 250 mg/kg and 500 mg/kg groups compared to the vehicle group. In the case of females, GGT, BUN, TG, HDL, and LDL levels were significantly (*p* < 0.05) increased in the treated group (Table 5). Although there were significant differences in treated rats (male; ALT, TP, D-BIL, female; TP, ALB) compared to the vehicle group, the data value was within the reference range (Lee et al., 2012 [28]). The kidney function parameters (urea, creatinine, and uric acid) did not reveal any relevant changes following BCP administration. No statistically significant differences in liver function parameters (ALT, AST, ALP) were noted (Table 5).

#### Histopathology

**FIGURE 3:**
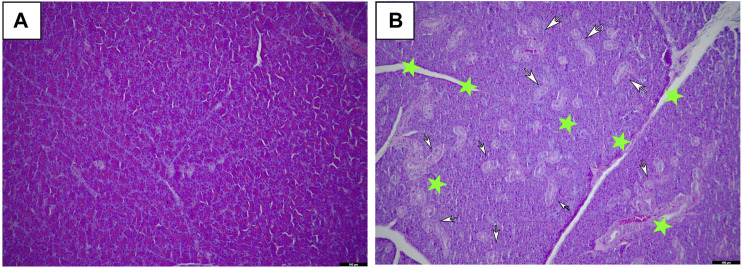
Histopathological changes of female SD rats in the 28-day oral administration toxicity test. **(A)** Normal microscopic structure of the pancreas of a female SD rat (x100). **(B)** Acinar cell atrophy (star) and ductular hyperplasia (arrows) in the pancreas of a female SD rat administered 1,000 mg/kg of Bromochlorophene (x100).

Macroscopic examination of the vital organs of treated animals revealed abnormalities in the color or texture when compared with the organs of the control group. The light microscopy examinations of the pancreas of the BCP treated group and the vehicle group rats are shown in Figure 3. Histopathological examination of the vehicle group and BCP treated rats showed abnormal structure and presence of any gross pathological lesion in organs. Acinar cell atrophy and ductular hyperplasia in the pancreas were clearly observed in the 1,000 mg/kg b.w. group (Figure 3).

### Genotoxicity

#### Bacterial Reverse Mutation Test

An Ames assay evaluated the mutagenicity of BCP, and no toxicity was observed (Table 6). Compared with the vehicle control, the number of reverted mutant colonies did not exceed two at all doses of each strain regardless of metabolic activity. Therefore, BCP was evaluated as a harmful substance that did not induce a return mutation under this test condition.

**TABLE 6:** Bacterial reverse mutation test.

Strain	Treated item	Revertant colonies/plate (Mean ± SD)			
		Dose (μg/plate)	Without S9-mix	Dose (μg/plate)	With S9-mix
TA98	BCP	0	24 ± 1.7	0	38 ± 3.1
		2.44	24 ± 3.5	2.44	38 ± 5.5
		4.88	27 ± 6.4	4.88	42 ± 1.2
		9.77	25 ± 2.1	9.77	42 ± 4.7
		19.5	32 ± 4.0	19.5	41 ± 5.0
		39.1	21 ± 3.2	39.1	32 ± 7.6
		78.1	0 ± 0.0	78.1	30 ± 5.6
Mutagenicity			Negative		Negative
TA100	BCP	0	123 ± 13.7	0	119 ± 8.7
		2.44	112 ± 7.0	9.77	123 ± 10.1
		4.88	118.15.6	19.5	134 ± 1.5
		9.77	122 ± 16.5	39.1	134 ± 4.2
		19.5	120 ± 16.5	78.1	119 ± 10.1
		39.1	72 ± 12.7	156	52 ± 8.7
		78.1	35 ± 5.7	313	19 ± 1.5
Mutagenicity			Negative		Negative
TA1535	BCP	0	10 ± 1.2	0	11 ± 1.7
		2.44	11 ± 1.7	9.77	12 ± 0.6
		4.88	12 ± 3.8	19.5	11 ± 2.9
		9.77	10 ± 1.5	39.1	11 ± 3.5
		19.5	8 ± 1.5	78.1	10 ± 1.7
		39.1	4 ± 0.6	156	3 ± 1.0
		78.1	2 ± 0.6	313	0 ± 0.0
Mutagenicity			Negative		Negative
TA1537	BCP	0	9 ± 2.5	0	13 ± 2.5
		0.61	8 ± 1.0	0.61	13 ± 3.5
		1.22	9 ± 2.0	1.22	20 ± 2.5
		2.44	9 ± 2.5	2.44	15 ± 1.0
		4.88	2.3	4.88	17 ± 1.2
		9.77	10 ± 2.5	9.77	16 ± 2.3
		19.5	5 ± 1.0	19.5	13 ± 3.5
Mutagenicity			Negative		Negative
WP2*uvr*A	BCP	0	35 ± 3.5	0	47 ± 3.8
		2.44	33 ± 1.0	9.77	43 ± 4.2
		4.88	41 ± 3.8	19.5	52 ± 4.6
		9.77	37 ± 2.9	39.1	48 ± 5.9
		19.5	38 ± 3.6	78.1	42 ± 5.6
		39.1	30 ± 3.1	156	31 ± 5.9
		78.1	20 ± 2.6	313	31 ± 7.9
Mutagenicity			Negative		Negative
Positive control					
TA98	2-NF	0.5	188 ± 12.1		
TA100	SA	1.5	648 ± 24.8		
TA1535	SA	1.5	456 ± 47.7		
TA1537	9-AA	80.0	287 ± 3.8		
WP2uvrA	4-NQO	0.5	238 ± 30.2		
TA98	B[a]P			1.0	318 ± 20.8
TA100	2-AA			1.0	99 ± 35.9
TA1535	2-AA			2.0	330 ± 4.4
TA1537	2-AA			2.0	224 ± 16.7
WP2uvrA	2-AA			10.0	557 ± 49.8

BCP, Bromochlorophene; 2-NF, 2-Nitrofluorene; SA, sodium azide; 9-AA, 9-Amino acridine; 4-NQO, 4-Nitroquinoline-1-oxide(4-NQO); B[a]P, benzo[a]pyrene; 2-AA, 2-Aminoanthrancene; SD, standard deviation.

#### *In vitro* Mammalian Chromosomal Aberration Test

Results of the *in vitro* chromosomal aberration assay are shown in Table 7. There was no statistically significant increase in the frequencies of aberrant metaphases with structural aberrations at any test article dose compared with the concurrent negative control. The positive control articles induced clear positive responses (*p* < 0.01).

**TABLE 7:** *In vitro* chromosome aberration test.

Treated item	Dose (μg/mL)	RPD(%)	No. of structural aberrant cells									No. of numerical aberrant cells			Others^a)^
			ctb	csb	cte	cse	frg	gap		Total (%)		end	pol	Total (%)	
								ctg	csg	gap-	gap+				
6 h Trt-18 h Rc (-S9)															
DMSO	0.0	100	1	1	0	0	0	0	0	2 (0.7)	2 (0.7)	0	0	0 (0.0)	0
BCP	2.5	98.0	Not observed												
	5.0	76.1	0	0	1	0	0	0	0	1 (0.3)	1 (0.3)	0	0	0 (0.0)	0
	10.0	69.3	0	0	3	0	0	0	0	3 (1.0)	3 (1.0)	0	1	1 (0.3)	0
	20.0	53.1	0	0	1	0	0	0	0	1 (0.3)	1 (0.3)	0	0	0 (0.0)	0
	40.0	36.4	Not observed												
MMC	0.1	58.7	8	0	67	1	0	0	0	^a^71 (23.7)	71 (23.7)	0	0	0 (0.0)	0
6 h Trt-18 h Rc (+S9)															0
DMSO	0.0	100	0	0	2	0	0	0	0	2 (0.7)	2 (0.7)	0	0	0 (0.0)	0
BCP	5.0	94.6	Not observed												
	10.0	93.8	0	0	0	0	0	0	0	0 (0.0)	0 (0.0)	0	1	1 (0.3)	0
	20.0	84.7	0	0	1	0	0	0	0	1 (0.3)	1 (0.3)	0	0	0 (0.0)	0
	40.0	75.7	0	0	0	0	0	0	0	0 (0.0)	0 (0.0)	0	2	2 (0.7)	0
	80.0	31.0	Not observed												
B[a]P	20	51.7	6	0	59	1	0	0	0	^a^63 (21.0)	63 (21.0)	0	1	1 (0.3)	0
24 h Trt-0 h Rc (-S9)															
DMSO	0	100	1	0	1	1	0	0	0	3 (1.0)	3 (1.0)	0	0	0 (0.0)	0
BCP	1.25	95.2	Not observed												
	2.50	97.0	2	0	0	0	0	0	0	2 (0.7)	2 (0.7)	0	0	0 (0.0)	0
	5.00	87.4	0	0	0	1	0	0	0	1 (0.3)	1 (0.3)	0	1	1 (0.3)	0
	10.0	47.9	1	0	0	0	0	0	0	1 (0.3)	1 (0.3)	0	0	0 (0.0)	0
	20.0	33.6	Not observed												
MMC	0.1	52.6	8	0	103	0	0	0	0	^a^108 (36.0)	108 (36.0)	0	0	0 (0.0)	0

BCP, Bromochlorophene; DMSO, dimethyl sulfoxide; ctg, chromatid gap; csg, chromosome gap; ctb, chromatid break; cte, chromatid exchange; csb, chromosome break; cse, chromosome exchange; frg, fragmentation; end, endoreduplication; pol, polyploidy; MMC, mitomycin C; B[a]P, benzo[a]pyrene; RPD, relative population doubling; Trt-Rec time, treatment-recovery times; gap-, total number of cells with structural aberrations excluding gap; gap+, total number of cells with structural aberrations including gap. a), Others were excluded from the number of cells with chromosomal aberrations.

a Significant difference from negative control by Fisher’s exact test; *p* < 0.01.

#### *In vivo* Mammalian Erythrocyte Micronucleus Test

There were no animals with any clinical signs or unscheduled deaths. The frequency of MNPCE was not increased at all dose levels of BCP, while MMC induced a clear increase in the frequency of MNPCE (*p* < 0.01). The ratios of PCE/(PCE + NCE) were not significantly different at all dose levels of BCP (Table 8).

**TABLE 8:** *In vivo* micronucleus test.

		Body weight (h after dosing)			
Treated item	Dose (mg/kg)	0	24	MNPCE/4000 PCE	PCE/(PCE+NCE)
BCP	0	33.86 ± 1.37	34.20 ± 1.37	0.02 ± 0.03	30.36 ± 2.75
	62.5	33.68 ± 1.17	33.63 ± 1.16	0.02 ± 0.03	29.32 ± 4.19
	125	33.85 ± 1.19	33.59 ± 1.23	0.05 ± 0.02	33.96 ± 3.19
	250	33.84 ± 1.32	33.65 ± 1.28	0.05 ± 0.02	32.1 ± 2.38
Positive control					
MMC	2	33.69 ± 1.53	33.65 ± 1.56	6.74 ± 0.85^a^	25.48 ± 3.37

BCP, Bromoclorophene; MMC, mitomycin C; PCE, polychromatic erythrocyte; NCE, normochromatic erythrocyte; MNPCE, PCE with one or more micronuclei.

a Significant difference from vehicle control group at *p* < 0.01.

### Dermal Application Study

**FIGURE 4:**
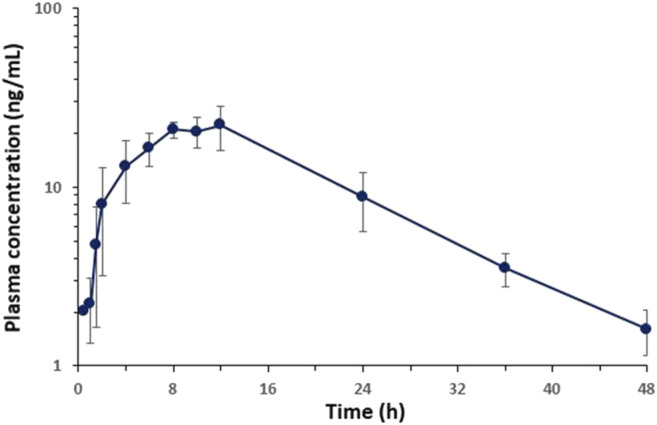
Average plasma concentration-time profiles of BCP vs. time obtained after topical application of hydrogel containing 0.1% of BCP in rats (*n* = 5).

The plasma concentration-time profiles of BCP after topical dermal application in rats are shown in Figure 4. Upon topical application, BCP was quantifiable in the plasma samples (30 min–1.5 h). After the initiation of topical application, plasma concentrations reached C_max_ within 8–12 h. The non-compartmental pharmacokinetic parameters of BCP are summarized in Table 9. After applying the hydrogel formulation, the elimination half-life (t1/2) was 10.35 ± 2.07 h, and topical bioavailability was 29.25 ± 6.09%. As a result, the half-life of the disappearance rate of BCP was significantly longer.

**TABLE 9:** Kinetic parameters of BCP in rats after topical dermal application.

Parameters	TD
T_1/2_ (h)	10.35 ± 2.07
T_max_ (h)	10.80 ± 1.79
C_max_ (ng/ml)	23.82 ± 5.29
AUC_all_ (ng h/ml)	470.09 ± 104.51
AUC_inf_ (ng h/ml)	494.59 ± 103.00
Vz/F (L/kg)	8.32 ± 2.75
CL/F (ml/min/kg)	9.10 ± 1.61
MRT (h)	18.31 ± 1.96
F (%)	29.25 ± 6.09

## Discussion

The usage of chemical products increases every year, and new chemical substances are distributed through household products. In addition to the rapid development of chemical products, interest in the safety of these household products is also increasing (Bolinius et al., 2018). According to the notification, these substances including solvent cleaner, rinsing products, sterilizing preservatives, sunscreen, blind spot substances, and non-intended exposure substances are stipulated in “Regulations of Safety Standards for Cosmetics” and “Standards and Specification for Hygiene Products” by the Ministry of Food and Drug Safety (MFDS) in Korea (MFDS (The Ministry of Food and Drug Safety), 2017a; MFDS, 2019).

Based on the solubility test results, BCP was suspended at a concentration of 1,000 mg/ml and was tested at a concentration of 1,000 mg/ml as the highest dose in the study of single-dose toxicity. There was no significant effect on pathological and clinical changes observed between the vehicle and all treatment groups. Therefore, the investigators concluded that the ALD of orally ingested BCP was higher than 1,000 mg/kg b.w. in SD rats. According to Loomis and Hayes (Wallace Hayes et al., 2020), substances with an LD_50_ between 5,000 and 15,000 mg/kg b.w. are considered as harmless.

Furthermore, the investigators tested three battery tests for BCP, which were the mammalian chromosomal aberration test, bacterial reverse mutation test, and mammalian erythrocyte micronucleus test, to check *in vivo* and *in vitro* genotoxicity, respectively (Azqueta and Dusinska, 2015; Marques et al., 2015; Madia et al., 2020). BCP showed negative results in all three genotoxicity studies. In these respects, the investigators concluded that BCP is a non-genotoxic substance.

In the study of toxicokinetics, the delay in the half-life of BCP during dermal application administration is presumed to be due to flip-flop pharmacokinetics, where the elimination rate constant is faster than the absorption rate constant. This is a common phenomenon of the drug concentration–time mechanism (Yáñez et al., 2011), which appears because the absorption rate of BCP exposed to the skin is slower than the rate of disappearance of treatment. The prolonged half-life of BCP dermal application exposure means that it can accumulate in the body at a higher concentration than other routes.

In the repeated-dose 28-day oral toxicity test, there was a significant difference in action metamorphosis between the vehicle and treatment groups. In BCP-treated rats, blood fat-related indicators T-CHO and TG were increased. Also, acinar cell atrophy and ductular hyperplasia in the pancreas were clearly observed in 1,000 mg/kg b.w.-treated female rats and acinar cell atrophy was dose-dependently observed in 500 and 1,000 mg/kg b.w.-treated female rats. The marked difference was seen in pancreas lesions associated with increased T-CHO and TG, which histological investigations in the existing study have supported. Hormones play a leading part in regulating blood cholesterol levels in relation to promoting metabolism in the body. It is well known that lipases promote lipolysis, reduce blood cholesterol and triglycerides, and maintain a normal blood lipid profile (Grandjean et al., 2000; Lee and Jun 2009; Larsson et al., 2014). Moreover, pancreatic juice contains at least three lipolytic enzymes: the well-known lipase, cholesterol ester hydrolase, and phospholipase (Wang et al., 2019). Therefore, abnormalities in the secretion of lipolytic enzymes increase blood lipid-related indicators and affect pancreatic lesions. The pancreas is an essential organ in nutrient metabolism, and malnutrition is closely related to pancreatic disorder. Patients with pancreatic deficiency show a disordered pattern of fat mass and lean body mass (Haaber et al., 2000). According to Henrik et al., 34% of patients with moderate to serious weight loss were found to have reduced lean body mass. Thus, this phenomenon may lead to decreased pancreatic functional ability (Rasmussen et al., 2013). Thus, food and water consumption increased, but there was no difference in the rate of weight gain, which can be explained as an abnormality in pancreatic function. The mortality rate was 85% at a 1,000 mg/kg b.w. dose in male rats. However, in other groups, no association was found between hematological changes (ALT, TP, T-CHO, D-BIL, HDL) and histological changes, and no histopathological changes were observed in 500 mg/kg-treated male rats. Overall, we can assume that the NOAEL is 250 mg/kg b.w. for 28 days for both female and male rats. Future investigations require a review of lipase measurement and male and female hormone metabolism.

## Conclusion

In summary, the authors conducted a toxicity test regarding genotoxicity, dermal application, and general oral toxicity. These toxicity study data revealed that T-CHO and TG affect the pancreas as targets of repeated-dose oral toxicity. Thus, NOAEL in this study is 250 mg/kg b.w. for female and male rats based on the increased CHO, TG, and observed pancreas lesions. The following outcomes indicated that this study might be utilized as a platform for scientific evidence by amassing toxicity information and producing toxicity data of the single or repeated-dose toxicity of BCP on household chemicals. Because there was no information of toxicity on BCP in humans, further study will be required for ensure its safe application in household products.

## Data Availability

The raw data supporting the conclusions of this article will be made available by the authors, without undue reservation.
